# Addressing social media platforms’ influence on academic research

**DOI:** 10.1057/s41599-026-06690-6

**Published:** 2026-02-13

**Authors:** Raffael Heiss, Isabelle Freiling

**Affiliations:** 1 Management Center Innsbruck, Innsbruck, Austria; 2 University of Utah, Salt Lake City, UT USA

**Keywords:** Cultural and media studies, Science, technology and society

## Abstract

Social media platforms play a central role in shaping today’s information ecosystem, yet access to both their internal data and even publicly visible content remains tightly restricted for academic researchers. This stands in sharp contrast to other industries, such as food and pharmaceuticals, where researchers can independently study product ingredients and effects. As a result, academic research on social media faces an unprecedented dependency on industry-controlled data, increasing the risk of bias and potentially distorting the evidence needed for effective regulation and policymaking. Drawing on research from other disciplines, we examine how industry influence operates and how researchers’ reliance on platforms for data may amplify industry influence. We identify four challenges in collaborations between researchers and social media platforms: restricted data access, selective funding, hard-to-detect influence, and institutional entanglements. These challenges risk undermining the independence and transparency of research in a field of growing societal relevance. Addressing these challenges requires policymakers to regulate data access, as illustrated by the EU’s Digital Services Act (DSA), which mandates data access for vetted researchers while safeguarding user privacy. In addition, new independent funding mechanisms could help ensure that research agendas remain free from platform interests. In parallel, the social science community must adopt stronger ethical standards and invest in “research on research” to detect and mitigate potential biases in policy-relevant research. With a dual approach—policy reforms and critical academic debates—we can ensure that research on social media platforms serves the public interest rather than platform priorities.

## Introduction

Social media platforms have emerged as powerful agents shaping today’s information ecosystem through algorithmic amplification and structural control over content distribution (Nielsen & Ganter 2022). At the same time, these platforms are under increasing scrutiny for contributing to systemic risks, including the spread of misinformation, rising polarization, and potential harm to users’ mental health (Allen et al. 2024; Kubin and Sikorski 2021; U.S. Surgeon General 2023).

Yet, researchers face significant barriers in studying these risks, as platforms rarely provide access to crucial data on their algorithms, content flows, or engagement dynamics (Krause et al. 2025). This lack of access to social media data has led to ongoing academic debates, such as the one sparked by Jonathan Haidt’s book *The Anxious Generation* (Haidt 2024). Haidt argues that social media is driving an increase in mental health issues among young people. Conversely, other researchers contend that there is insufficient data to substantiate these claims (Odgers 2024). Unfortunately, both sides in the debate are limited by the platforms’ control over the data needed to properly assess social media’s impact (Davidson et al. 2023; de Vreese and Tromble 2023).

## Data access as an entry point for industry influence

Due to this limited access to data, some academic researchers have begun collaborating with social media platforms. However, this makes academic research vulnerable to industry bias—a phenomenon well documented in the pharmaceutical, tobacco, and food industries (Bero 2022; Fabbri et al. 2018; Oreskes and Conway 2010). What makes the situation with social media platforms unique is that these companies hold exclusive access to the data. In other industries, academics can often generate their own data for independent research. This unprecedented control over data not only heightens the risk of familiar biases – such as those tied to funding – but also grants platforms greater influence over the data they release and the academic alliances they form.

An example of platform influence is the partnership between academic researchers and *Meta* to examine the role of Facebook and Instagram in the 2020 US presidential election (Wagner 2023). Some findings of this partnership suggested that algorithmic changes affected what users saw but had minimal impact on attitudes or polarization (Guess et al. 2023; Nyhan et al. 2023). *Meta* emphasized this interpretation, issuing a statement that the studies added to “a growing body of research showing that there is little evidence that key features of *Meta’s* platforms alone cause harmful ‘affective’ polarization or have meaningful effects on key political attitudes, beliefs, or behaviors” (Meta, 2023). This frame was echoed in media reports, headlining that changing *Meta’s* algorithm may not solve the problem (e.g., The Washington Post 2023).

This is not to criticize the academic researchers, who implemented safeguards to minimize platform influence, including appointing an independent rapporteur (Wagner 2023). Nonetheless, the exclusive access to data granted by *Meta* limited opportunities for replication and raised questions about transparency and potential bias. These concerns were reinforced when it was later revealed that Facebook made significant algorithm changes during the study period (Thorp and Vinson 2024). The changes made may have decreased polarizing content, and the study’s authors acknowledged that these adjustments could have affected the results. This not only raises concerns about the independence of scientific research but also has broader policy implications. For instance, *Meta* funds the *American Edge Project*, a policy advocacy group that could leverage research results to lobby against regulatory oversight (Wheeler 2022).

We argue that such collaborations are also entry points for other potential biases, which have been extensively examined in other fields (Fabbri et al. 2018). We reflect on the lessons learned from other industries and discuss how they can be applied to social media platforms.

## Lessons learned from other industries

First, meta-analyses in other fields comparing studies with and without industry funding show that industry support can bias policy-relevant research results. A striking example is a study on the effects of sugar-sweetened beverage consumption on overweight and type 2 diabetes (Schillinger et al. 2016), which found that most industry-sponsored studies reported no effect, while nearly all independent studies identified positive associations. The phenomenon of reporting industry-favorable results is also observed in other domains, including pharmaceutical and tobacco research (Barnes and Bero 1998, Bero, 2018, Gardner and McMahon 2007; Lundh et al. 2018).

Another important lesson is that industry influence can be subtle. Even small financial relationships or gifts can introduce bias, affecting research designs that are often considered resistant to such biases, like randomized controlled trials (Schillinger et al. 2016). This subtlety can be explained by reciprocity theory, which suggests that small gifts may create a sense of indebtedness, prompting researchers to unconsciously reciprocate with favorable behavior (Katz et al. 2010).

The next lesson is that industry influence can be exerted at any stage of the research cycle—whether through the research question, study design, data collection, analysis, or interpretation of results. This influence often begins early in the process, for instance, by shaping the research agenda through targeted funding calls and the selection of research topics and questions. Funding in particular can serve as an agenda-setting tool, prioritizing topics that align with industry interests, for example, emphasizing individual responsibility rather than industry practices (Bero, 2022; Fabbri et al. 2018).

Finally, industry players often seek to institutionalize their influence by creating or funding non-profit organizations and research centers that produce work aligned with corporate goals. A notable example is the *International Life Science Institute (ILSI)*, a global research network sponsored by *Coca-Cola* and other major food companies. *ILSI* has faced heavy criticism for its attempts to strategically influence scientific debates and for promoting biased policy recommendations, such as emphasizing exercise over dietary change to combat obesity (Greenhalgh 2019; Mialon et al. 2021).

## Challenges in industry-academy collaborations for social media

The lessons outlined above should be carefully considered when evaluating partnerships with social media platforms. Drawing on these lessons, we identify four fundamental challenges that need to be addressed (see Table 1). These challenges are not mutually exclusive, but they intersect and can thus influence and reinforce one another.

**Table 1:** Overview of key challenges in industry-academy collaborations for social media, lessons learned from other industries, and avenues for action.

Challenges	Lessons	Avenues for Action
**1**. Restrictive data access for independent researchers.	*No direct equivalent in other industries.*	• Ensure low-threshold access for independent researchers. • Adjust existing regulations and establish new agencies to oversee data access for independent research.
**2**. Platforms selectively fund and collaborate with researchers to address policy-relevant research questions.	Industry influence can bias policy-relevant research results.	• Limit industry collaborations to topics that do not present a conflict of interest, e.g., research on technological innovations. • Conduct systematic reviews and meta-analyses to examine the impact of industry funding and collaboration on research outcomes.
**3**. Even when influence is suspected, platform influence is difficult to detect and prove.	Influence can be subtle and can be exerted at any stage of the research process.	• Eliminate formal and informal dependencies (personal contact with funders). • Raise awareness of researchers’ susceptibility to bias and develop ethical guidelines. • Establish truly independent agencies to manage and distribute funds for independent research.
**4**. Platforms establish long-term collaborations and funding schemes, creating permanent dependency.	Industry players seek to institutionalize their influence on science.	• Critically discuss institutionalized industry influence through intermediary organizations within the scientific community. • Examine potential biases associated with research tied to these organizations.

The **first challenge** is restrictive data access, which is unique to social media platforms. Imagine if researchers were denied access to the ingredients of food products or drugs to study their effects on human health. However, when it comes to social media products, access to their “ingredients,” such as algorithms and input data, is limited. This is problematic because some of these “ingredients” may pose risks, and most countries lack agencies dedicated to overseeing them (Persily and Tucker 2021). In the United States, for example, the *Food and Drug Administration* (FDA) ensures the safety of drugs and food products. By contrast, no equivalent body exists to review the safety of social media products.

Limiting data access is not only an issue with *Meta*’s platforms. *YouTube* and *TikTok*, for instance, have been even more restrictive (Persily and Tucker 2021). Such restrictions are a powerful force – not only because they leave consumers in the dark, but also because they compel researchers to collaborate with social media companies, since abstaining from this field is not an option. In Europe, the *Digital Services Act* (DSA) represents a promising step forward by requiring platforms to grant academic researchers access to relevant data. This enables independent investigations into the risks of platform content and features and obliges platforms to act on identified harms.

The **second challenge** is that platforms selectively fund researchers and topics to address policy-relevant research questions, which can bias the evidence for policy-making. The phenomenon of big tech funding social science research is relatively new, meaning that many social scientists are inexperienced in dealing with industry entanglements. Raising awareness of potential conflicts of interest is therefore crucial. It should be noted, however, that industry sponsorship can also be fruitful, for example, when partnering on topics that are less politically sensitive and drive technological innovation.

The **third challenge** is that even when influence is suspected, it is difficult to detect and to prove. Outside researchers do not have access to the private conversations and decisions made at each step of the research process. In current models of industry-academy collaboration, platforms can, besides selecting researchers to work with, define the scope of data and metrics shared (Wagner 2023). For example, platforms can define concepts in a certain way and share only the data that matches their definitions. This allows platforms to influence the data that is made available, the research questions and designs that can be applied, and limit the ways in which the data can be analyzed.

Research collaborations with platforms are sometimes legitimized by employing open science practices, including pre-registration of hypotheses and analysis plans (Wagner 2023). However, influences can be subtle: the provision of resources and access to data, or even personal collaboration, can induce feelings of reciprocity that may subconsciously affect researchers. Furthermore, open science practices may protect some, but not all, aspects of the research cycle. For instance, even pre-registered studies can involve bias due to industry influence on the research questions or data access.

The **fourth challenge** is that platforms institutionalize their influence by establishing long-term collaborations. For instance, *Meta* funds researchers globally through early-career fellowships. In 2018, *Meta* also established an institutional collaboration with *Social Science One*. The idea was to provide better access to *Meta’s* internal data, but researchers have struggled from the start to get access to the promised data (Shirin 2021). More recently, the *Chan Zuckerberg Initiative* funded a new artificial intelligence institute at *Harvard* with $500 million (Kahn and Levien 2021). Another example is *Jigsaw*, *Google’s* technology incubator, which also funds academic research activities (e.g., Roozenbeek et al. 2022).

While some institutional partnerships may indeed offer valuable opportunities for technological progress, strategic interests may operate in the background when research touches on policy-relevant questions. For instance, whistleblowing from inside *Meta* indicates that platforms may bury internal research that points to negative platform effects on users’ health (Klar and Shapero 2024). This also calls into question whether research funding from large platforms is purely philanthropic. While promising for the development of new technologies, these activities may also introduce bias into policy-relevant research questions. They thus need to be closely and critically monitored by the scientific community.

## Avenues for addressing the challenges

The challenges outlined above can be addressed through targeted policy interventions and a more critically engaged social science community. We suggest how this can be done below, recognizing that their concrete implementation will involve value-based decisions that may differ across stakeholders and contexts.

Policymakers should strengthen regulatory frameworks to ensure that independent researchers can access both internal and publicly available platform data to evaluate systemic risks without industry interference. Of course, access to platform data can pose privacy risks, as users may be re-identified through simple search queries. Any data access mechanism must therefore be accompanied by robust safeguards to protect user privacy (Krause et al. 2025).

Europe’s DSA is a first step in this direction. Article 40 of the DSA requires very large online platforms to provide vetted researchers with access to internal data, including public communication data (such as posts and comments), user account metadata, and data governance information, such as algorithmic selection and testing mechanisms (Klinger and Ohme 2023). This access is intended to enable independent assessments of systemic risks—such as the spread of harmful misinformation or the impact of platform features. To ensure user privacy and data security, the DSA mandates strict vetting procedures and requires that all data access comply with applicable data protection laws (Klinger and Ohme 2023).

To support implementation, *National Digital Services Coordinators* monitor compliance and report to the *European Commission*. In addition, the DSA 40 Data Access Collaboratory initiative documents and evaluates the implementation of Article 40, focusing on how researchers and non-profit organizations can gain access to platform data (https://dsa40collaboratory.eu/). Unfortunately, early findings indicate that many platforms interpret eligibility and risk criteria too narrowly, reject or delay applications without clear justification, and fail to provide adequate documentation (Jaursch et al. 2024; Klinger et al. 2024). These findings clearly show how much of a challenge data access is even with first laws in place and raise the question of how the European Commission will respond.

While the DSA is limited to the EU, social media interactions span across the globe, which means similar regulation would be needed elsewhere as well. If implemented effectively, the DSA could serve as a model for other regions seeking to reconcile data access with user privacy and platform accountability.

Additionally, it is important to limit the opportunities for potential platform influence, which can be achieved by establishing clear ethical guidelines and by limiting the points of contact between funding agents and independent researchers. Policymakers should prioritize independent research funding, particularly in areas prone to conflicts of interest. One approach is to provide more resources for independent research, for example, through national science funds. Another is to establish independent agencies that collect and distribute industry or platform funds and manage calls for proposals and decisions on which project will be funded. Those agencies could then help ensure that research objectives, data access, and outcomes remain unbiased.

Regulatory measures alone will not be sufficient. The social science community must also exercise greater oversight of industry activities, following the example set by other disciplines. Researchers need to critically examine how existing industry collaborations may have influenced research outcomes, particularly in areas where platforms may have a stake (e.g., impact of platforms on political polarization, misinformation, or users’ health). To address these concerns, more “research on research” is needed, such as systematic reviews and meta-analyses that compare the topics and results of studies with and without industry engagement (Bero 2018, 2022).

Together, more effective regulations and a critical community of social science researchers committed to examining potential industry bias will lay a strong foundation for reducing these risks and ensuring the validity of social science research in an increasingly technology-dominated era.

## Data Availability

No datasets were generated or analyzed during the current study.
